# Engine performance and emissions evaluation of surfactant-free B30 biodiesel–diesel/water emulsion as alternative fuel

**DOI:** 10.1038/s41598-023-37662-4

**Published:** 2023-06-30

**Authors:** Mohamad Qayyum Mohd Tamam, Md Reashed Tasvir Omi, Wira Jazair Yahya, Ahmad Muhsin Ithnin, Hasbullah Abdul Rahman, Md. Mujibur Rahman, Hasannuddin Abd Kadir, Hirofumi Noge, Tsuyoshi Koga, Chungpyo Hong, Takeshi Otaka, Eiji Kinoshita

**Affiliations:** 1 Advanced Vehicle System, Malaysia-Japan International Institute of Technology, Universiti Teknologi Malaysia, Jalan Sultan Yahya Petra, 54100 Kuala Lumpur, Malaysia; 2 RTES Technology (M) Sdn. Bhd., Jalan Kebun, 41000 Klang, Selangor Malaysia; 3 College of Engineering, Universiti Teknologi MARA, Cawangan Johor, Kampus Pasir Gudang, 81750 Masai, Johor Malaysia; 4 Graduate School of Education, Okayama University, 3-1-1 Tsushima-naka, Kita-ku, Okayama 770-8530 Japan; 5 Graduate School of Science and Engineering for Innovation, Yamaguchi University, 2-16-1 Tokiwadai, Ube City, Yamaguchi 755-8611 Japan; 6 Department of Mechanical Engineering, Kagoshima University, 1-21-24 Korimoto, Kagoshima City, Kagoshima 890-8580 Japan

**Keywords:** Biodiesel, Mechanical engineering, Diesel fuel

## Abstract

Malaysia is one of the top exporters of palm oil, and although currently facing fierce resistance towards palm oil imports in some parts of the globe, one of the ways to utilize this commodity is by increasing palm biodiesel content in local commercial diesel. However, due to the oxygen-rich nature of biodiesel, its utilization suffers from increased nitrogen oxides (NO_x_) emission compared to conventional diesel. To mitigate this issue and improve diesel engine performance and emissions using biodiesel–diesel blends, this study attempted to investigate implementation of a real-time non-surfactant emulsion fuel supply system (RTES) which produces water-in-diesel emulsion as fuel without surfactants. NO_x_ reducing capability of water-in-diesel produced by RTES has been well documented. Therefore, in this study, 30% biodiesel–diesel (B30) was used as the base fuel while B30-derived emulsions consisting of 10 wt%, 15 wt% and 20 wt% water content were supplied into a 100 kVA, 5.9-L common rail turbocharged diesel engine electric generator. Fuel consumption and exhaust emissions were measured and compared with commercially available Malaysian low grade diesel fuel (D2M). Evidence suggested that emulsified B30 biodiesel–diesel produced by RTES was able to increase brake thermal efficiency (BTE) up to a maximum of 36% and reduce brake specific fuel consumption (BSFC) up to 8.70%. Furthermore, B30 biodiesel–diesel emulsions produced significantly less NO_x_, carbon monoxide and smoke at high engine load. In conclusion, B30 biodiesel–diesel emulsions can be readily utilized in current diesel engines without compromising on performance and emissions.

## Introduction

Malaysia is one of the leading exporters of palm oil and is able to produce its own palm oil biodiesel. However, as the European Union (EU) is becoming increasingly hostile towards palm oil imports^1,2^, Malaysia need to find ways to fully utilize this commodity in the domestic market. One of the most promising ways to increase palm oil utilization is by increasing palm oil-based biodiesel content in the domestic diesel. Since 2010, Malaysia have raised the percentage of biodiesel content in domestic diesel fuel from 5 to 10 vol%. The 12th Malaysia Plan (RMK-12) has set a goal to impose 30 vol% of biodiesel–diesel blend (B30) in domestic diesel fuel by the year 2025^3^. As of now, commercially available diesel sold in service stations nationwide consist of either 7 vol% (B7) or 10 vol% (B10) biodiesel–diesel. Nonetheless, such efforts triggers concern among engine manufacturers as well as commercial and private vehicle owners as to how this implementation impacts fuel costs and efficiency, as well as engine performance and durability.

Biodiesel production cost is higher as compared to conventional petroleum-based diesel fuel^4^, therefore increments of biodiesel content in biodiesel–diesel fuel blends will result in rising fuel production costs. Domestically in Malaysia, the government has for decades imposed a large-scale subsidy program on fuel prices^5^ which may be able to absorb the price hike of increasing biodiesel–diesel fuel blends, thereby stabilizing the market price of commercial diesel. Nonetheless, as biodiesel content in biodiesel–diesel fuel increases, the overall calorific value per volume shall reduce due to biodiesels possessing lower calorific value (CV) than petroleum-based diesel. This shall lead to increased fuel consumption as more fuel shall be consumed in order to produce the same amount of energy as conventional petroleum-based diesel. Furthermore, fuel consumption could also increase as biodiesel possess higher viscosity which could lead to poor fuel pumping and spray behavior^6^.

Biodiesel in general possess higher kinematic viscosity and density than conventional diesel^7^. These factors affect fuel droplets atomization and entrainment when injected into the combustion chamber. However, in a modern diesel engine equipped with a common rail fuel injection system, it is argued that the effects of the aforementioned properties were rather insignificant. This is because, high pressure fuel injection introduced by the common rail fuel injection system enables improved fuel droplets atomization and evaporation, therefore enhancing combustion process^8^. Nonetheless, biodiesel emit higher nitrogen oxides (NO_x_) emissions when taking into account longer ignition delays caused by higher peak combustion temperatures as the result of higher oxygen content in biodiesel^9^. Furthermore, due to lower CV of biodiesel, fuel consumption is considerably higher compared to conventional diesel. Several research have documented higher fuel consumption when using various biodiesel blends in common rail injection diesel engines^10–12^.

In order to reduce NO_x_ emissions and improve fuel consumption of biodiesel and biodiesel–diesel blends, there is a need to reduce in-cylinder temperatures and improve combustion efficiency. One strategy to achieve this is by using Water-in-diesel (W/D) emulsion fuel. The effects of W/D has been studied for many years and has shown promising improvements in terms of engine performance and exhaust emissions^13–16^. This can be attributed to micro-explosion of micro-sized water droplets dispersed in diesel oil. Micro-explosion occurs during combustion when water droplets suspended in W/D emulsions undergoes explosion due to it having lower boiling point. This results in a secondary atomization of the primary fuel spray, decreasing distribution of fuel droplets, resulting in improved combustion^17,18^. It was reported that as water content in W/D emulsion increases up to a maximum of 20%, Brake Thermal Efficiency (BTE) increases while gas temperature (EGT) behaved conversely, indicating lower peak temperatures due to the charge cooling by water evaporation^15^. In another study, soot emission was significantly by 50% by using W/D micro-emulsions containing 3.5 vol% of water^19^. In relation to biodiesel–diesel W/D emulsions, it was documented that W/D can reduce exhaust gases such as carbon monoxide (CO), unburned hydrocarbon (UHC), and soot opacity, while carbon dioxide (CO_2_) emissions increased^20^. The authors inferred that the CO_2_ increases observed are related to a more complete combustion, as well as hydroxide (OH) radicals present during water vaporization assists formation of CO_2_ from CO.

Nonetheless, since water and oil could not be mixed naturally, synthesis of W/D emulsions require the use of a chemical additive known as surfactant or emulsifier to suspend the water particles in the diesel oil for a sustained period of time. Despite its benefits, surfactants used in W/D emulsions are known to cause fuel filters clogging by displacing deposits in the fuel lines and fuel tanks^21^. Another shortcoming of surfactants is due to its expensive nature, large-scale production of W/D emulsion fuels would not be a viable substitute to petroleum-based diesel, rendering commercialization difficult^22^.

To remove the dependence on surfactants to produce W/D emulsion fuel, Ithnin et al.^23^ developed a device capable of producing W/D emulsion fuel without addition of any surfactant by incorporating a real time emulsifying device which mixes diesel and water within the fuel line and on-demand to the engine. It operates by using a high shear mixer in combination with an ultrasonic agitator to produce the W/D emulsion. This device was named Real-Time Non-Surfactant Emulsion Fuel Supply System; or in short, RTES. It was initially tested on a 5 kW single cylinder mechanical fuel injection diesel engine producing 6.5 wt% W/D emulsion fuel and the results proved that engine BTE improved by 3.59%, while Brake Specific Fuel Consumption (BSFC) was reduced by 3.89%. Exhaust emissions also showed favorable improvements, with NO_x_ and particulate matter (PM) emissions plunged by 31.7% and 16.3% respectively when compared to conventional diesel.

Moving forward, further research has been done to examine the effects of RTES implementation on diesel engines. In general, RTES implementation was effective in lowering down NO_x_ and smoke emissions with simultaneous increases in BTE as well as reductions in fuel consumption. A summary of previous research on the effects of W/D produced by RTES towards various engine applications are explained in Table 1.

**Table 1:** Summary of past research on the effects of W/D produced by RTES.

Application of RTES	Engine Type	Test Fuel	Test Condition	Findings													Reference
				Droplet size	Sediment time	ν	HRR	BTE	FC	ICP	EGT	CO	UHC	NO_x_	Smoke	PM	
Engine dyno	1-cylinder, 0.4 L, direct injection	D2M 5% W/D2M 5% W/D2M with emulsifier	Constant speed: 3000 rpm Variable load: 1,2,3,4 kW	0.41 μm–15.38 μm	25 s			↑	↓					↓		↓	^17^
		B5 5% W/B5 10% W/B5 Steam W/B5	Constant speed: 2000 rpm Variable load: 1,2,3,4 kW			↑	↑			↓		↑	↑	↓		↓	^27,28^
		D2M W/D2M	Variable load: 1,2,3,4 kW									↑		↓		↓	^29^
		D2M W/D2M-S W/D2M-T W/D2M-R	Variable load: 1,2,3,4 kW	9.63–18.59 μm					↓	≈		↑		↓		↓	^30^
Diesel-electric generator	1-cylinder, 0.418 L, direct injection	D2M 3% W/D2M 6% W/D2M 9% W/D2M	Constant speed: 3200 rpm Variable load: 1,2,3,4 kW						≈		↓	↑		↓	≈		^31^
	6-cylinder, 5.9L, common rail direct injection,	D2M 3% W/D2M 6% W/D2M 9% W/D2M 15% W/D2M 20% W/D2M	Constant speed: 1500 rpm Variable load: 20,40,60,80%					↑	↓			↑	↓	↓	↓		^32^
Lorry with mechanical injection	4-cylinder, 2.8 L, direct injection	7–10% W/B10	Vehicle idling Constant speed: 1500 rpm Variable load: 20,40,60,80%					↑	↓	↓		↑		↓			^26^
		D2M 16.6% W/D2M	WVU-5 peak						↓			↑		↓			^33^
		D2M 5% W/D2M 6.5% W/D2M 10.8% W/D2M 30% W/D2M	WVU-5 peak						↓		↓	↑		↓	↓		^34^
Sports Utility Vehicle	4-cylinder, 2.2L, common rail direct injection,	D2M 6.5% W/D2M	WVU-5 peak (modified)						↓		↓			↓	↓		^35^
Industrial burner	Dual stage heavy oil pressure jet burner	D2M 5% W/D2M 10% W/D2M 15% W/D2M	–						↓			↓	↓	↓		↓	^36^

However, one of the key elements of the original RTES design was the role of ultrasonic agitator as one of the mixing methods, which reduced overall energy efficiency due to its high-power requirement. To ensure smooth and stable supply of W/D emulsion to the engine, the ultrasonic agitator demands 120 W of electrical energy^23^. In addition, a study to determine RTES durability during extended use reported ultrasonic agitator failure after 26 h^24^.

In reaction to this, further design improvements were conducted RTES Technology (M) Sdn. Bhd. which eliminated the use of ultrasonic agitator from the mixing method^25^. The updated design consists of static mixers and booster pumps to facilitate fluid turbulence and promote mixing. This design concept was tested by Mahdi et al.^26^ and it was found that when 7–10 vol% of water content in W/D emulsion were mixed at 3500 rpm for 1 min, stability was maintained within 128 s. In the same study, pilot tests on a mechanical injection-type diesel engine showed lower NO_x_ and fuel consumption, implying that W/D emulsions produced using this design exhibit similar qualities to the original RTES design.

This study is a continuation of our research on RTES-produced W/D emulsions as alternative fuel in industrial diesel-electric generators^32^. In this paper, the effect of B30 emulsion fuel (B30E) produced using the updated RTES design was evaluated. The main purpose of this study focuses on examining the effects of B30 emulsions with variable water contents towards lowering NO_x_ emissions often associated with biodiesel–diesel blends. Secondly, since previous studies on RTES were conducted using only naturally aspirated mechanical-type fuel injection diesel engines with conventional diesel and/or low biodiesel–diesel blends (B10), it is important to establish the performance and emissions profile of a modern common rail injection-type engines using higher biodiesel–diesel W/D blends; in this case B30, to examine the readiness of RTES implementation should B30 rollout be carried out nationwide according to RMK-12 by 2025.

## Experimental details

### Test fuels

Base fuel used in this study is Malaysian Euro 2M low grade diesel fuel (D2M). It is commercially available in domestically in Malaysia and contain 10 vol% palm oil Fatty Acid Methyl Ester (POME) off the shelf. Therefore, since this study aims to investigate the effects of B30 biodiesel–diesel W/D blends, another 20 vol% of POME was added to D2M to form B30. The specifications of D2M and POME are indicated in Table 2. During preparation, 20 vol% of POME was measured and added to D2M before it is mixed using high shear mixer at a constant speed of 500 rpm in a closed container, to ensure mixture homogeneity. B30 was then immediately fed to the engine where it was let to run until the fuel lines were filled with B30 before any test run was conducted.

**Table 2:** Physicochemical properties of Malaysian Euro 2M low grade diesel (D2M) and palm oil based (POME).

Properties	Unit	ASTM test method	D2M	POME
Calorific value (CV)	MJ/kg	D240	43.8	40.1
Density at 15 °C	g/mL	D1298/D4052	0.837	0.877
Kinematic viscosity at 40 °C	cSt	D445/D7042	3.4	4.5
Flash point	°C	D93-A	68	174
Sulphur	ppm	D4294/D2622	320	400
Cloud point	°C	D2500/D5772	13	18
POME content	vol%	D7371	10	100

Meanwhile, to produce W/D emulsions of B30 (B30E), domestic tap water was used as the dispersed phase of the emulsion. The properties of tap water are explained in Table 3. Water percentages considered in this study was 10% (B30E10), 15% (B30E15), and 20% (B30E20). Higher water content was not desirable as higher than 20% will result in excessive vibration and engine stall. Meanwhile, physicochemical properties of B30 were not tested due to the need to continuously mix B30 to prevent coalescence and separation between D2M and POME.

**Table 3:** Physicochemical properties of Malaysian domestic tap water ^27^.

Properties	Unit	Value
Density at 25 °C	g/mL	1.0241
Specific conductivity at 25 °C	μS/cm	0.0532
Dynamic viscosity at 25 °C	cSt	0.902
Vapour pressure at 20 °C	mmHg	17.4
Isothermal compressibility at 0 °C	Vol/atm	46.4 × 10^6^
Surface tension at 0 °C	Dyne/cm	72.74
Specific heat at 17.5 °C	J/g°C	3.898
Temperature of maximum density	°C	− 3.25
Freezing point	°C	− 1.91

**Figure 1:**
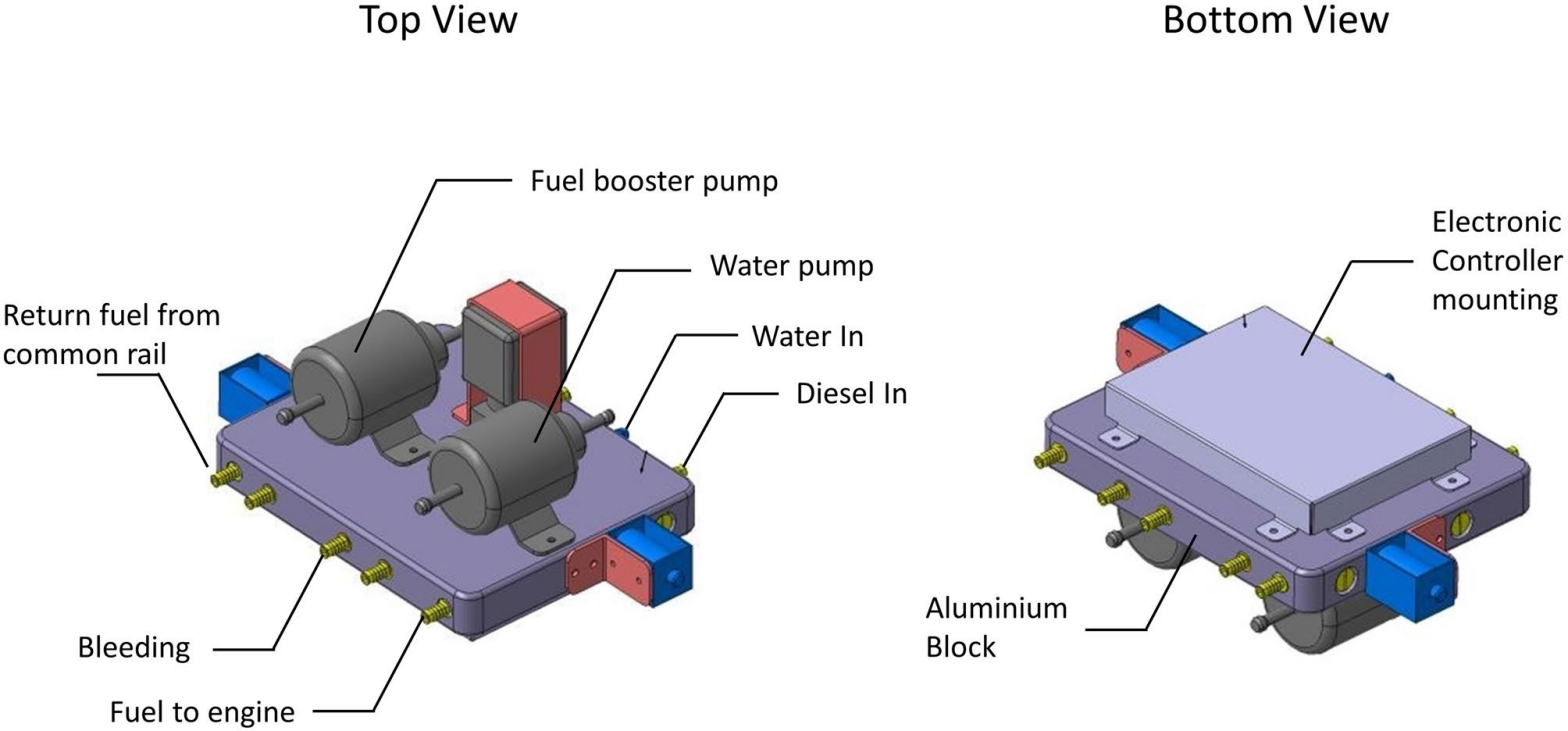
Updated RTES design.

W/D emulsions were produced by RTES using the updated RTES design developed by RTES Technology Sdn. Bhd. by removing ultrasonic agitator. Current RTES design incorporates a turbulence inducing mixing conduit and booster pumps. Figure 1 illustrates the updated RTES design. Detailed design was not revealed by the technology developer, however further information related to RTES can be acquired from their website^25^.

### Engine testing

**Figure 2:**
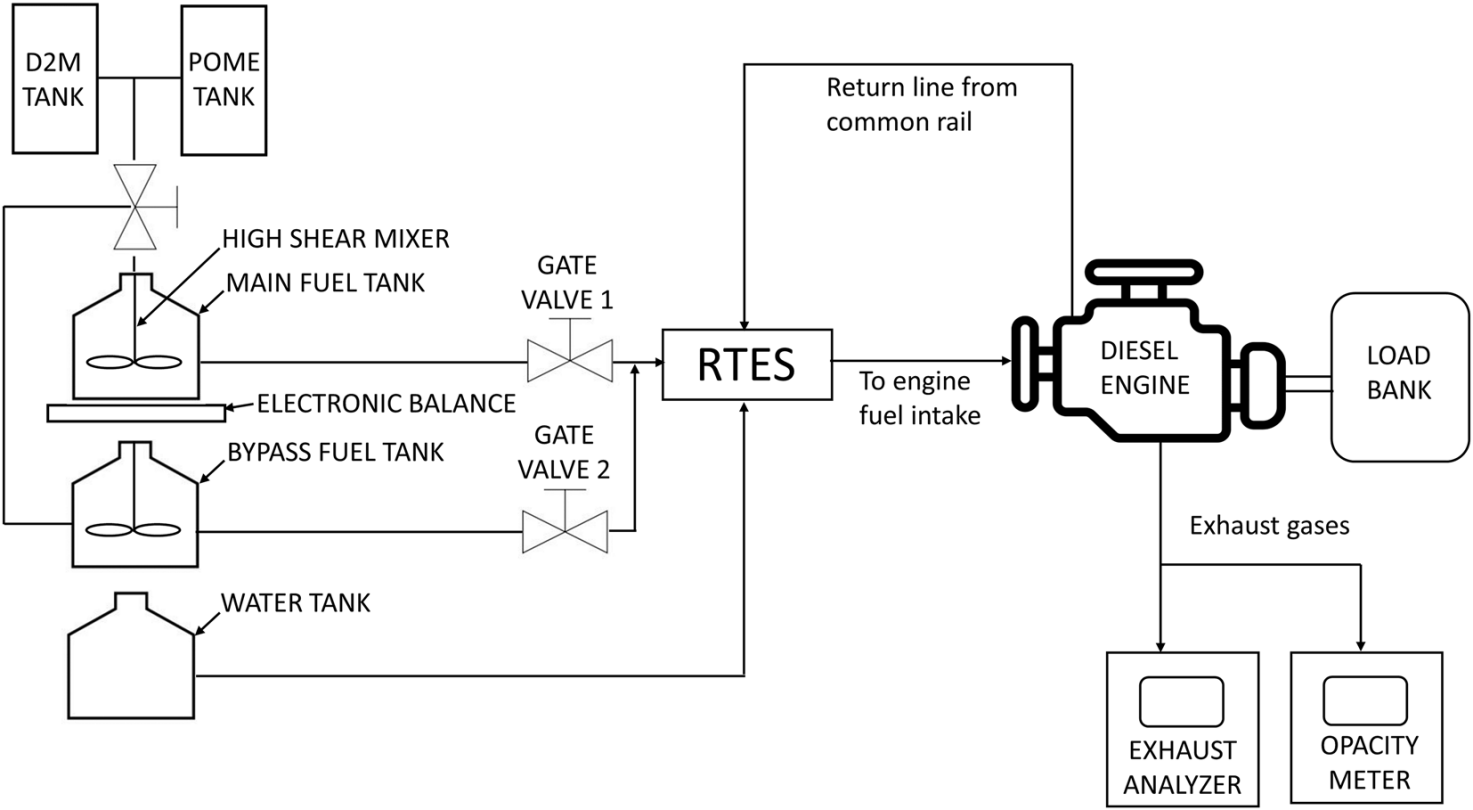
Schematics of engine testing setup using RTES.

**Figure 3:**
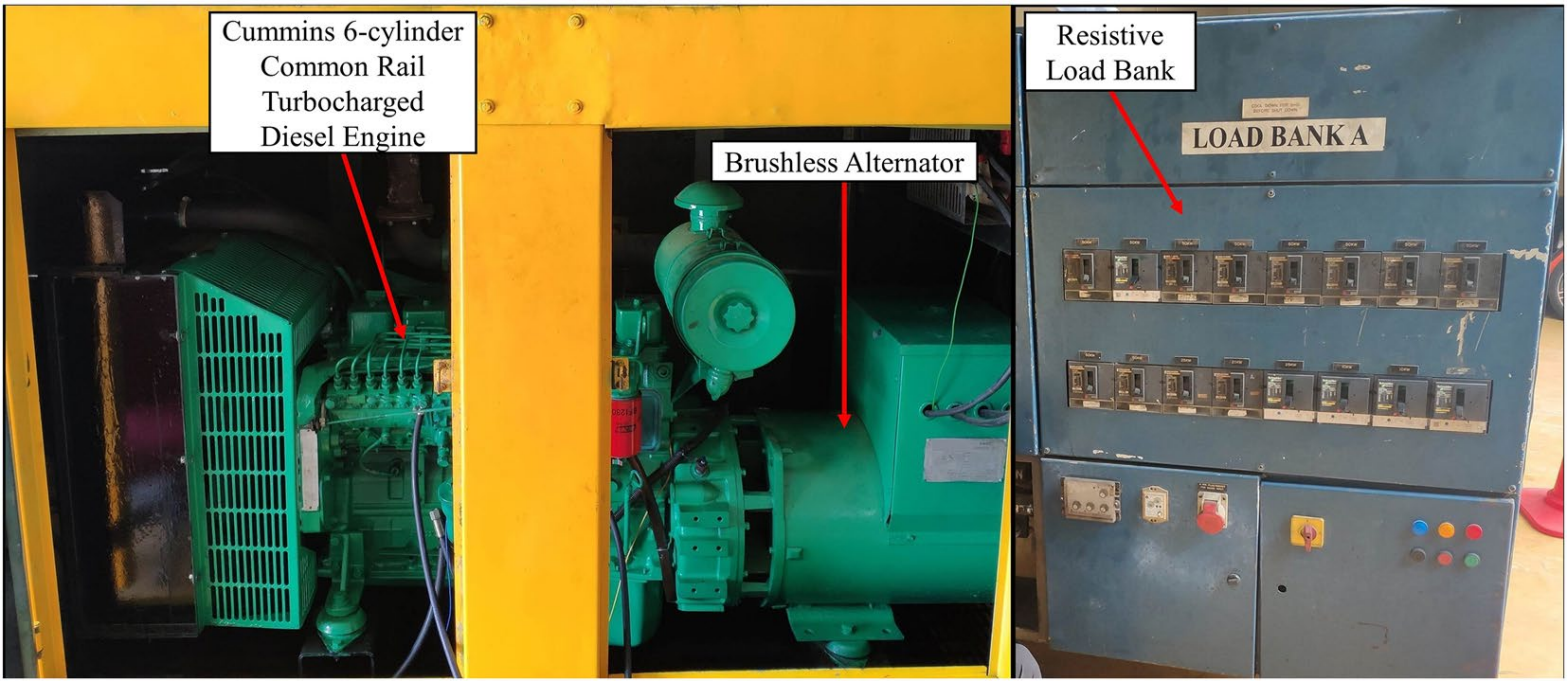
Test engine and load bank configuration.

Figure 2 depicts the engine testing setup for this study. Tests were done on a 5.9 L, 6-cylinder, turbocharged induction diesel engine with common rail injection. The engine is connected to a 100 kVA 4-pole 3-phase AC electric generator maintained at a constant speed of 1500 rpm to produce 420 V. The diesel-electric generator specifications are depicted in Table 4. Tests were conducted under variable electrical loads condition ranging from low load (5 kW), medium load (34 kW) and high load (64 kW) provided by an electrical load bank. The diesel-electric generator and load bank used in this study are shown in Fig. 3. The load bank is resistive-type, and the loading verification was done in-house according to Eq. (1)^37^ where power factor is assumed as 1 due to resistive loads. Thus, the results of the loading verification are explained in Table 5.

**Table 4:** Specifications of diesel-electric generator.

Parameter	Specification
Make/Model	Cummins/6BT5.9-G2
Configuration	Cast iron, in-line, 6 cylinder
Bore × Stroke (mm)	102.1 × 119.9
Displacement (L)	5.9
Compression ratio	17.3:1
Aspiration	Turbocharged
Fuel injection system	Common rail direct injection
Standby power (kW)	92
Prime power (kW)	86

**Table 5:** Electrical loading verification.

Load (%)	Hz	Engine speed (rpm)	Voltage (V)			Current (A)			Active power (kW)			% Power difference		
			L1	L2	L3	L1	L2	L3	L1	L2	L3	L1	L2	L3
0	52.5	1564	418	418	417	–	–	–	–	–	–	–	–	–
25	51.6	1538	417	418	418	29.7	29.4	29.1	21.45	21.29	21.07	0.23	1.00	2.01
50	50.5	1505	418	418	418	63.2	63.0	62.2	45.76	45.61	45.03	6.41	6.07	4.73
75	49.7	1481	418	418	416	82.9	82.7	81.6	63.49	63.35	62.25	1.56	1.78	3.48
100	48.4	1444	418	418	418	108	108	107	78.19	78.19	77.47	9.08	9.08	9.92

1 $$Active power=\sqrt{3}\times Linevoltage \left(V\right)\times Linecurrent \left(A\right)\times power factor$$

As indicated by the manufacturer data in Table 4, peak power of the diesel-electric generator was achieved at 86 kW, therefore it is considered as 100% load. In relation to loading verification explained previously in Table 5, at 100% loading, an error between 9.08 and 9.92% was found. Meanwhile, at 75% (64 kW) loading and below, a maximum error of 6.41% was evaluated. Hence, for the purposes of this study, tests were conducted to a maximum load of 64 kW to ensure a stable power output with minimal error.

**Figure 4:**
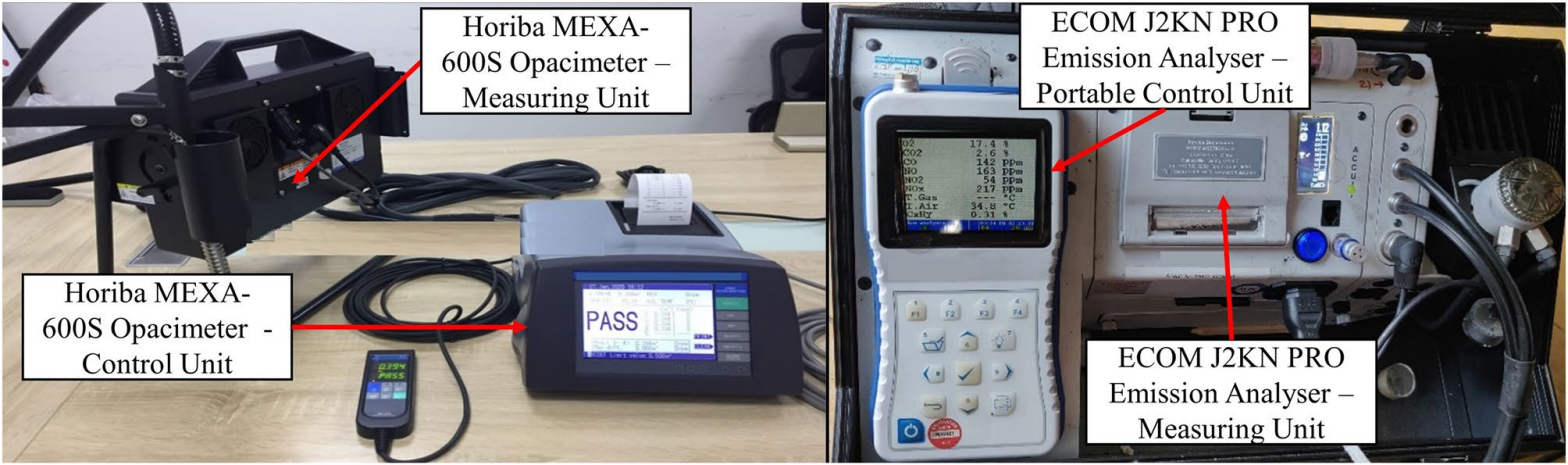
Exhaust gas analysis measuring equipment.

For engine performance and emissions testing, the engine is first warmed up using B30 until lubricant temperature stabilizes at approximately 60 °C. Following that, gate valve 1 is closed and gate valve 2 as shown in Fig. 2 is opened simultaneously, and the weight of main fuel tank is measured using electronic balance (accuracy ± 0.001 kg). This functions to bypass the main fuel tank by redirecting fuel flow to another fuel source. Upon completion of weighing, gate valve 1 is re-opened and gate valve 2 is re-closed and at the same time RTES system is activated to allow emulsification of B30 and water. Resultant B30E emulsion fuels are fed into the engine and tested for 6 min cycles under each load condition. This procedure is repeated for several cycles. Fuel consumed is measured by calculating the difference in weight prior to RTES activation and after each test cycles concluded. In each test cycle, NO_x_ and CO exhaust gas emissions are measured using ECOM J2KN PRO gas analyzer while exhaust smoke opacity is measured using HORIBA MEXA-600S opacimeter. Both measuring equipment are depicted in Fig. 4. Technical specifications of both the gas analyzer and opacimeter are explained in Tables 6 and 7 respectively.

**Table 6:** Specifications of J2KN PRO gas analyzer.

Measurement	Range	Accuracy	Resolution
Carbon Monoxide (CO)	0–10,000 ppm	± 2% measured	1.0 ppm
Carbon Monoxide (high)	0–63,000 ppm	± 2% measured	5.0 ppm
Nitric Oxide (NO)	0–5000 ppm	± 5% measured	1.0 ppm
Nitric Oxide (low)	0–500 ppm	± 5% measured	1.0 ppm
Nitrogen Dioxide (NO_2_)	0–1000 ppm	± 5% measured	1.0 ppm
Nitrogen Dioxide (low)	0–100 ppm	± 5% measured	0.1 ppm
Unburned Hydrocarbon (C_x_H_y_)	0–4.00 vol%	± 5% measured	0.01 vol%

**Table 7:** Specifications of HORIBA MEXA-600S opacimeter.

Parameter	Description
Measuring principle	Opacity method (Photo sensor detector)
Range	Opacity: 0.00 to 100% Light absorption coefficient: 0.000 to 10.00 m^−1^
Accuracy	± 2%
Sampling method	Partial flow
Test standards	ISO-11614

### Experimental and uncertainty calculations

In this study, BTE and BSFC were calculated from fuel consumption data. Equation (2) ^38^ was used to evaluate BSFC:

2 $$BSFC=\frac{{\dot{m}}_{fuel}}{{P}_{b}}$$

where ṁ_fuel_ is the fuel mass flow rate measured in (g/h) while P_b_ is the engine brake power in kW. On the other hand, Eq. (3)^38^ was used to calculate BTE.

3 $$BTE,{\eta }_{thermal}=\frac{3600}{BSFC\times CV}\times 100$$

where BSFC is obtained from Eq. (2) measured in (g/kWh), and CV for D2M and POME verified in-house using CAL2K ECO bomb calorimeter. Meanwhile, CV for B30 was calculated by adding the CV of POME to D2M to achieve 30:70 blend ratio for biodiesel–diesel (42.9 MJ/kg). Furthermore, to calculate the CV for B30E10, B30E15, and B30E20, weighted average method was used, as explained in Eq. (4) ^39^.

4 $${CV}_{B30E}=\frac{\left({CV}_{B30}\times {mass}_{B30}\right)+\left({CV}_{water}\times {mass}_{water}\right)}{{mass}_{B30E}}$$

where CV_water_ is zero.

Furthermore, the calculation for uncertainty analysis used in this study is as shown in Eq. (5) ^40^.

5 $${\omega }_{R}=\sqrt{{\left(\frac{\delta R}{\delta {x}_{1}}{\omega }_{1}\right)}^{2}+{\left(\frac{\delta R}{\delta {x}_{2}}{\omega }_{2}\right)}^{2}+{\left(\frac{\delta R}{\delta {x}_{3}}{\omega }_{3}\right)}^{2}+\dots +{\left(\frac{\delta R}{\delta {x}_{n}}{\omega }_{n}\right)}^{2}}$$

where ω_R_ is the total uncertainty of the experimental data, while ω_1_, ω_2_, ω_3_, to ω_n_ represent independent variables. This equation is used to calculate the uncertainty of BTE and BSFC which consisted of independent variables such as ṁ_fuel_, P_b_, and fuel CV as evident in Eq. (3). For instance, uncertainty of BSFC is given by Eq. (6) ^41^.

6 $${\omega }_{BSFC}=\sqrt{{\left(\frac{\delta BSFC}{\delta {\dot{m}}_{fuel}}{\omega }_{{\dot{m}}_{fuel}}\right)}^{2}+{\left(\frac{\delta BSFC}{\delta {P}_{b}}{\omega }_{{P}_{b}}\right)}^{2}}$$

Therefore, the overall uncertainty of the experiments is as explained in Eq. (7)

7 $${\omega }_{Overall}=\sqrt{{uncertainty\, of\left(BSFC\right)}^{2}+{\left(BTE\right)}^{2}{+\left(CO\right)}^{2}{+\left(N{O}_{x}\right)}^{2}{+\left(Smoke\right)}^{2}} =\sqrt{{uncertainty\, of\left(1.38\right)}^{2}+{(1.38)}^{2}{+(2.28)}^{2}{+(3.87)}^{2}{+(2.63)}^{2}} = \pm 5.56\mathrm{\%}$$

## Results and discussion

### Brake thermal efficiency (BTE)

**Figure 5:**
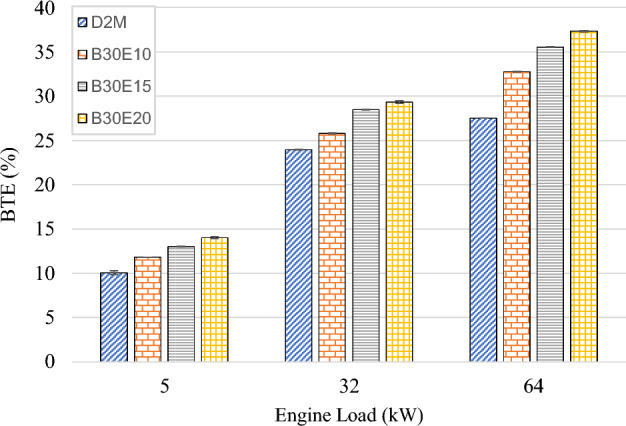
BTE of test fuels at different engine loads.

Figure 5 shows the effects of W/D emulsions towards engine BTE when operated under variable loads. It is obvious that BTE increases as engine load increases for all test fuels. At high engine load, D2M achieved 27.5% BTE while B30E10, B30E15 and B30E20 show significantly higher BTE at 32.8%, 35.6% and 37.4% respectively. B30E20 achieved the highest efficiency overall with a sizeable increase of 36.0% when compared to D2M at high engine load. Furthermore, at all tested engine loads, it was found that as water percentage in B30E emulsions increases, BTE increased as well. There are four postulations for this major improvement. Firstly, it may be due to micro-explosion effect that occurs when water droplets inside the emulsion fuel evaporated and tear the fuel droplet apart which can further improve combustion and thus producing higher in-cylinder pressure^42,43^. Although common rail fuel injection can produce very fine fuel droplets during pre-combustion, secondary atomization from the evaporation of water inside the B30E emulsion fuels still can occur and contribute towards enhancing air–fuel mixture. Secondly, emulsion fuels introduced into diesel engines tend to prolong ignition delay, which increases time for air–fuel mixing and evaporation, therefore improving combustion quality^17,44,45^. Thirdly, it may be due to the presence of oxygen in B30 biodiesel. Even though B30 possess lower calorific value when compared to D2M, which leads to increased fuel consumption as evident from previous documented research^10–12^, it does not mean that the combustion process itself was not efficient. The presence of oxygen in biodiesels assist combustion at fuel rich regions, therefore improving combustion efficiency. However, in most cases the improvement of combustion efficiency could not offset the deficiency of energy content within the biodiesel fuel itself and hence resulted in higher BSFC. Finally, it is possible that evaporation and expansion of water droplets within the emulsion fuel during combustion process increased the overall rate of heat release when compared to conventional diesel. Water do not carry any energy for the purpose of combustion, and act only as an expansion agent within the combustion cylinder by absorbing the heat released during combustion. As the content of water (as the expansion agent) increases, the higher is the rate of heat release inside the combustion cylinder. In fact, in-cylinder pressure could be higher as compared to conventional diesel if the start of ignition can be modified to be at the same timing^30,46^. In contrast, if there is too much water in the emulsion fuel, the absorption of heat by water particles will start to quench some part of the chemical reactions occurring during combustion, therefore limiting the maximum water content that could be introduced into the emulsion fuel. In this study, it was found that 20 wt% of water was the optimum water percentage within the W/D emulsion that can act as the expansion agent without significantly affecting chemical reaction of combustion process.

### Brake specific fuel consumption (BSFC)

**Figure 6:**
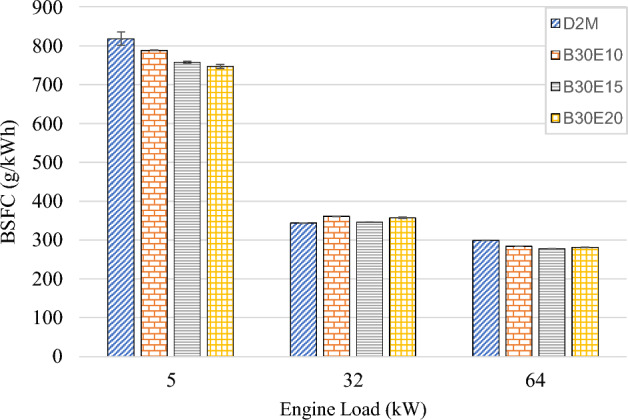
BSFC of test fuels at different engine loads.

In this study, for the purpose of calculating BSFC, only B30 is considered as the fuel. This is because water is not a combustible substance. This method was chosen with reference to previous studies on BSFC behavior of W/D emulsion fuels^28,32,47^. Therefore, in accordance with Eq. (2), BSFC of D2M and various water percentage B30E when subjected to increasing engine loads are as illustrated in Fig. 6. It is observed that BSFC decreases as the engine load increases. This signifies that engine combustion efficiency is enhanced at higher engine loads^17^. Furthermore, it is also observed that at each tested engine load, BSFC improved as water percentage in B30E increases. Most notably at low engine load, where BSFC for B30E10, B30E15 and B30E20 fuels showed significant decrease by 3.67%, 7.43% and 8.70% respectively, as compared to D2M. Meanwhile, at high engine load of 64 kW, B30E15 displayed maximum BSFC reduction with a 7.19% improvement. Considering all engine loads, average reduction of each B30E emulsion fuels is 1.18%, 4.63% and 3.6% for B30E10, B30E15 and B30E20 respectively. This observed reduction of BSFC can be considered as significant, since a previous report observed a marginal BSFC increase when B30 biodiesel was fueled in a common rail injection diesel vehicle as opposed to B10^10^. Despite having a calorific value deficit of about 16.8% when compared to D2M, significantly lower BSFC and higher BTE achieved with B30E15 showed that micro explosion effect in W/D emulsion is sufficient to overcome the same engine loads with lower amount of fuel.

### NO_x_ emissions

**Figure 7:**
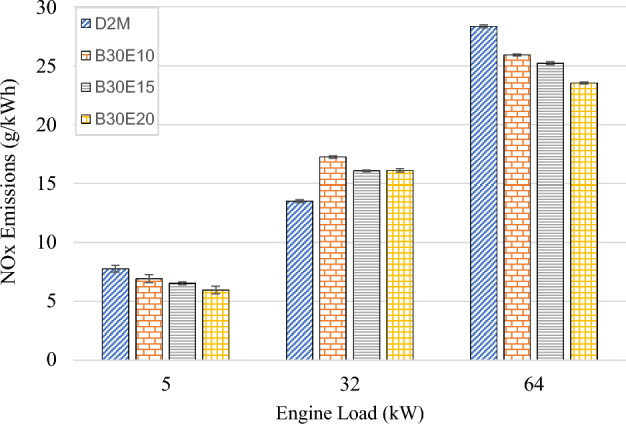
NO_x_ emission of test fuels at different engine loads.

NO_x_ emissions of D2M and B30E under increasing engine loads are as shown in Fig. 7. It is obvious that NO_x_ emission increases as engine load increases. At the low load engine condition of 5 kW, the average NO_x_ emission is at the minimum level of 60.4 g/kWh. As engine load increases, average NO_x_ emissions increase by measures of approximately 400% and 950% for medium and high loads respectively. NO_x_ gases are formed typically at high temperatures exceeding 1800 K through Zeldovich mechanism^48^. Therefore, as engine load increases, combustion cylinder temperature increases, resulting in a higher rate of NO_x_ formation. Furthermore, as water content in B30E increases, NO_x_ emissions decrease at all tested engine loads. Particularly at high load, D2M emitted 672 g/kWh of NO_x_ while B30E10, B30E15 and B30E20 emulsions produced lower NO_x_ by margins of 5.95%, 5.65% and 11.6% respectively. Water droplets present in B30E fuels resulted in lower flame temperatures during combustion as the result of latent heat absorption by water particles. Hence, NO_x_ formation by Zeldovich mechanism was restricted^49^. In addition, as water content in B30E increases, the amount of B30 fuel injected per volume is considerably lower, reducing the amount of combustion by-products, and further limiting NO_x_ formation^42^. Moreover, as water percentage increases, chances of more oxygen molecules ionize to form of hydroxyl (OH) radicals increases significantly, leading to lower NO_x_ formation^29^. Nonetheless, reduction of NO_x_ observed in this study is rather minimum when compared to previous studies^17,22,34^. This observation could be explained for two reasons. Firstly, B30E emulsions contain higher biodiesel content which translates to higher oxygen content in the fuel. Therefore, even though it is expected that B30 should produce higher NO_x_ emissions (due to oxygen presence), since water droplets are present in B30E, formation of NO_x_ was suppressed. Secondly, it is possible that due to higher combustion temperature promoted by common rail fuel injection coupled with a turbocharged induction air intake system, micro explosion of minute water particles in B30E emulsions could not effectively reduce combustion temperature in a magnitude observed in previous studies. These assertions were substantiated in a separate study which documented that a common rail turbocharged engine fueled with polyoxymethylene dimethyl ethers-diesel, a highly oxygenated fuel, was unable to effectively suppress NO_x_ formation under various engine loads^50^.

## CO emissions

**Figure 8:**
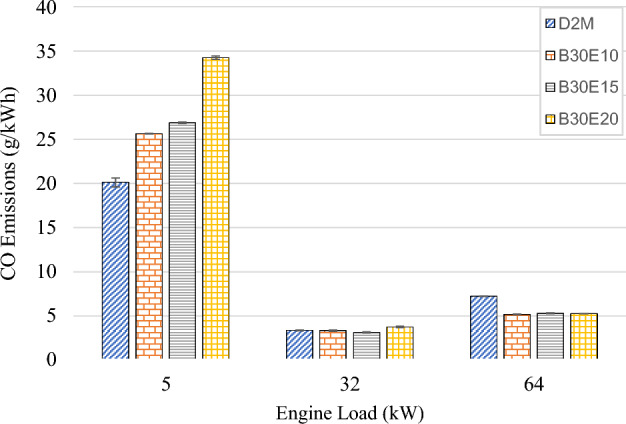
CO emission of test fuels at different engine loads.

Figure 8 shows the CO emissions of D2M and B30E under various loads. It is evident from the figure that CO emissions for all fuels are at the highest at low engine load. This is due to lower combustion cylinder temperature as the result of increase in heat loss per cycle^51^. Therefore, the rate of oxidation from CO to CO_2_ below 1400 K decreases^17^. Meanwhile, a slight increase is observed at high engine load, where CO emissions for all test fuels are slightly higher as compared to medium load. It can be explained that, at higher engine load, excess fuel is injected into the combustion cylinder which lead to stratified rich mixture regions. This in turn results in lesser contact between fuel and oxygen, leading to poor combustion within these regions. This notion was corroborated by a previous study that reported CO emissions increase in higher engine loads due to oxygen deficiency at the end of fuel jet impinged on the cylinder wall^52^. Nonetheless, it is obvious that at the low engine load, B30E emulsions displayed substantially higher CO emissions as compared to D2M. This is due to cooling effect which occurs during combustion of W/D emulsion, which encouraged incomplete oxidation of CO to CO_2_ in the presence of water droplets^51,53^. However, at high engine load B30E emulsions showcased much lower CO emissions than D2M. Higher engine loads are characterized by higher in-cylinder temperatures and pressures, where it can be argued that micro explosion occurs more violently, resulting in finer fuel droplets distribution, ultimately improving combustion^17^.

### Smoke opacity

**Figure 9:**
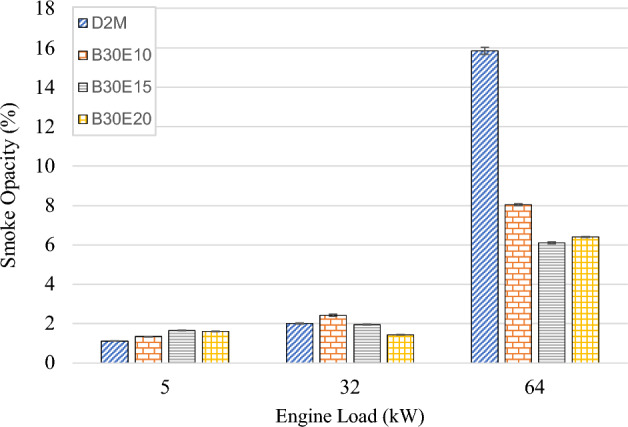
Smoke opacity of test fuels at different engine loads.

Figure 9 illustrates smoke opacity profile of D2M and various B30E under increasing engine load. It is clear that smoke opacity increases as engine load increases. This is because, more fuel is injected into the engine in high engine loads, causing air–fuel ratio to decrease, hence resulting in formation of smoke due to incomplete combustion^54^. Generally, it can be seen at high engine load, smoke opacity becomes lower as water content in B30 increases. In fact, maximum smoke opacity reduction is achieved by B30E15 under high loads, with a magnitude of 61.5% reduction with respect to D2M. Again, it is obvious that the role of micro explosion is very effective in improving fuel atomization, achieving higher combustion quality^22^ in high engine load environment characterized by higher in-cylinder pressure and temperature. Another contributing factor is due to the presence of higher OH radicals in B30E, air entrainment is enriched, further reducing smoke formation^55^. Similar trends were observed previously using D2M derived W/D emulsions where a maximum smoke opacity reduction of 87.0% was achieved by 21.8 wt% water content when fueled into the same test engine^32^. In addition, this trend is also observed for other types of biodiesel–diesel emulsion such as Nerium biodiesel–diesel, which showed up to 12.96% reduction in smoke opacity^56^, attributed by the effects of micro explosion. It can also be noted that the presence of oxygen in POME (hence B30) contributed towards more efficient combustion and suppressed formation of smoke^57^ In short, RTES implementation has been effective in reducing smoke emissions in common rail injection engines, which has negated suspicions that the effect of micro explosion is less pronounced in high pressure fuel spray of common rail injection. Furthermore, similar to CO emissions trend presented in Fig. 8, higher smoke emissions are detected in low engine load when fueled with B30E where the effect of micro explosion is less significant.

## Conclusion

This research attempted to improve performance and emissions of a 100 kVA common rail fuel injection, turbocharged diesel electric generator by using water-in-diesel emulsion fuel derived from higher biodiesel–diesel blends (B30) without addition of any emulsifier or surfactant. The base fuel chosen was a low-grade Malaysian Euro 2 diesel fuel (D2M) and three emulsified fuels of B30 produced by mixing D2M with POME (B30E). Water percentages considered were 10 wt% (B30E10), 15 wt% (B30E15) and 20 wt% (B30E20). B30E were produced using Real-Time Non-Surfactant Emulsion Fuel Supply System (RTES) immediately before entering the common rail fuel injection system.

The following conclusions are drawn from the experiment:

1. Brake Thermal Efficiency (BTE) at each tested engine load increased as water percentage in B30E emulsion fuels increased. The highest increment was observed at high engine load by B30E20 with 36.0% increment as compared to D2M.
2. Generally, BSFC reduced for all B30E emulsion fuels except for a slight increase at medium engine load, as compared to D2M. The maximum reduction was observed at low engine load with 8.70% reduction by B30E20. At high engine load, B30E15 showed a maximum reduction of 7.19%.
3. As water percentage in B30E emulsion fuels increased, NO_x_ emissions were reduced. However, the margin the reductions observed were not as pronounced as the ones observed in previous studies, possibly due to higher combustion temperatures when using forced induction common rail injection systems.
4. B30E emulsion fuels shows higher CO emission concentration at low engine load and lower CO emission concentration at high engine load than D2M. Combustion temperature at different engine loads influence the oxidation of CO to CO_2_ and strength of micro-explosion of B30E emulsion fuels.
5. Smoke opacity of B30E emulsion fuels reduced significantly as compared to D2M at high engine load. This proves that micro explosion restricted formation of soot particles by improving combustion through secondary fuel atomization. However, the effect of micro-explosion was weaker at low and medium engine loads.

In short, higher blends biodiesel–diesel W/D emulsions produced by RTES was able to improve engine thermal efficiency and fuel consumption as well as reducing NO_x_ and soot emissions when installed in a modern diesel engine. Based on the results of this study, RTES implementation is recommended when B30 biodiesel mandate is enforced in Malaysia in the near future by 2025.

## Data Availability

All data generated or analyzed during this study are included in this published article and available from the corresponding author based upon reasonable request.

## Abbreviations

AC: Alternate current
B10: 10 vol% biodiesel–90 vol% diesel
B30: 30 vol% biodiesel–70 vol% diesel
B30E10: 10 wt% water-B30 emulsion
B30E15: 15 wt% water-B30 emulsion
B30E20: 20 wt% water-B30 emulsion
B7: 7 vol% biodiesel–93 vol% diesel
BSFC: Brake specific fuel consumption
BTE: Brake thermal efficiency
CO: Carbon monoxide
CO_2_: Carbon dioxide
CV: Calorific value
D2M: Malaysian Euro 2M diesel
EGT: Exhaust gas temperature
EU: European union
FC: Fuel consumption
HRR: Heat release rate
ICP: In-cylinder pressure
NO_x_: Oxides of nitrogen
OH: Hydroxide ion
PM: Particulate matter
POME: Palm oil fatty acid methyl ester
RMK-12: 12th Malaysia plan
rpm: Revolutions per minute
RTES: Real-time non-surfactant emulsion fuel supply system
UHC: Unburned hydrocarbons
W/B5: Water-in-(5 vol% biodiesel–95 vol% diesel) emulsion
W/D: Water-in-diesel emulsion
W/D2M: Water-in-Malaysian Euro 2M diesel emulsion
W/D2M-R: Rainwater-in-Malaysian Euro 2M diesel emulsion
W/D2M-S: Seawater-in-Malaysian Euro 2M diesel emulsion
W/D2M-T: Tap water-in-Malaysian Euro 2M diesel emulsion

## List of symbols

${\dot{m}}_{fuel}$: Mass flow rate of fuel
$${P}_{b}$$: Brake power
vol%: Percentage by volume
wt%: Percentage by mass
μm: Micrometer
ν: Kinematic viscosity
